# Prognostic Factors and Treatment Results of High-Grade Osteosarcoma in Norway: A Scope Beyond the “Classical” Patient

**DOI:** 10.1155/2015/516843

**Published:** 2015-02-17

**Authors:** Kjetil Berner, Kirsten Sundby Hall, Odd R. Monge, Harald Weedon-Fekjær, Olga Zaikova, Øyvind S. Bruland

**Affiliations:** ^1^Department of Oncology, Oslo University Hospital, Norwegian Radium Hospital, 0424 Oslo, Norway; ^2^The Norwegian Cancer Registry, 0304 Oslo, Norway; ^3^Department of Oncology, Haukeland University Hospital, 5020 Bergen, Norway; ^4^Oslo Center for Biostatistics and Epidemiology, Research Support Services, Oslo University Hospital, 0424 Oslo, Norway; ^5^Department of Orthopedics, Oslo University Hospital, Norwegian Radium Hospital, 0424 Oslo, Norway; ^6^Institute of Clinical Medicine, University of Oslo, 0318 Oslo, Norway

## Abstract

*Purpose*. A retrospective study of prognostic factors and treatment outcome of osteosarcoma (OS) during modern chemotherapy era with focus on patients with primary metastatic disease, nonextremity localisation, or age >40 years (nonclassical OS). *Methods*. A nationwide cohort, comprising 424 high-grade Norwegian bone OS patients, was based on registry sources supplemented with clinical records from hospitals involved in sarcoma management between 1975 and 2009. *Results*. Only 48% were younger patients with tumour in the extremities and without metastasis at diagnosis (classical OS). A considerable discrepancy in survival between classical and nonclassical OS was observed: 61% versus 26% 10-year sarcoma specific survival. Twice as many of the former received both adequate surgery and chemotherapy compared to the latter. This could only partly explain the differences in survival due to inherent chemoresistance in primary metastatic disease and a higher rate of local relapse among patients with axial tumours. Metastasis at diagnosis, increased lactate dehydrogenase, age > 40 years, and tumour size above median value were all adverse prognostic factors for overall survival. *Conclusion*. We confirm a dramatic difference in outcome between classical and nonclassical high-grade OS patients, but treatment variables could only partly explain the dismal outcome of the latter.

## 1. Introduction

Multimodal treatment including multiagent chemotherapy has been essential to improve the survival of high-grade osteosarcoma (OS) patients [1–7]. The literature has predominantly focused on “classical OS” (COS); that is, extremity localized primary tumour, high-grade histology, age below 40 years, and no detectable metastasis at primary diagnosis. The prognoses are dismal for other subgroups of OS, that is, the “nonclassical OS” (NCOS) [5, 8–10]. Patients with axial OS may die due to lack of local control, even without detectable metastases. The chemoresistant disease in patients presenting with overt metastases is also an unsolved clinical challenge. The poor tolerance to adequate chemotherapy in the elderly represents another hurdle.

In this paper we report outcome of patients with NCOS during the modern chemotherapy era. Our cohort represents an unselected Norwegian OS population [10]. The purpose of this study was to compare patients with COS and NCOS with respect to patient characteristics and prognostic factors related to treatment outcome. To our knowledge only a few nationwide studies have previously been published [10–17]. None of these have specifically addressed a scope beyond the classical patient.

## 2. Material and Methods

### 2.1. Patient Cohort

**Figure 1:**
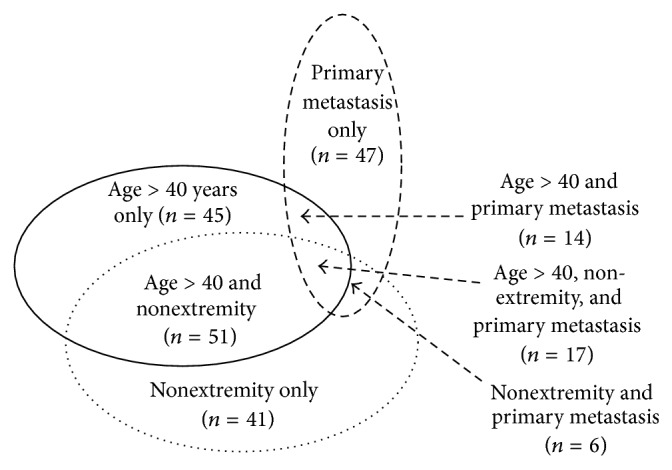
Nonclassical high-grade osteosarcoma (221 patients) with illustration of the overlaps between subgroups.

We have analysed 424 histologically verified high-grade bone OS patients diagnosed in Norway between 1975 and 2009 embracing all subgroups of NCOS (Figure 1), including secondary OS [10]. Variables relevant to this study were retrospectively validated based on multiple and partly overlapping data and registry sources supplemented with clinical records from all Norwegian hospitals involved in sarcoma management. Most patients were treated at the Norwegian Radium Hospital [10]. As expected, we have not reached full completeness regarding clinical information for all patients in the study (see Table 1), especially among patients diagnosed with OS during the 1970s. The database is located at the Norwegian Cancer Registry (NCR).

### 2.2. Demographic and Tumour Related Variable

All demographic and tumour related variables are presented in Tables 1-2.

*Tumour size* was measured from surgical specimens and/or radiographic images at diagnosis and defined as the maximum length of tumour in cm.* Duration of symptoms* was the interval in months between first symptom and time of biopsy. Unfortunately we could not dichotomise this interval into “patients delay” and “doctor's delay” [18] due to lack of accurate information in too many of the clinical case records. Median tumour size above 10 cm and median duration of symptoms longer than 3 months were defined as elevated values in the analyses.

*Serum Alkaline Phosphatase (ALP) and Serum Lactate Dehydrogenase (LDH).* Normal range, measured in international units at time of diagnosis, for ALP and LDH, was considered in line with the common Nordic Reference interval from May 2003 [19], that is, ALP: 0–17 years, <400 U/L; >17 years, <105 U/L; LDH: 0–10 years, <400 U/L; 11–70 years, <205 U/L; >70 years, <255 U/L. Analyses before May 2003 needed a 60 and 50% reduction in values, respectively, to be compatible with the above mentioned thresholds.

### 2.3. Treatment Variables

Before the introductions of the prospective clinical trials of the Scandinavian Sarcoma Group (SSG) in 1980 [5], several patients were treated according to the “CAMOS” regime [12]. During the study period, seven consecutive chemotherapy protocols were running: SSG II [20], SSG VIII [21], ISG/SSG I [22], ISG/SSG II [23], SSG XIV [24], Euroboss 1 [25], and the EURAMOS-1 [25–27], respectively. Patients not eligible for inclusion in the protocols were considered for individualized chemotherapy adjusted for age and toxicity.

The Euroboss 1 protocol still remains active in many European countries, and for the EURAMOS-1 only the results from the good responder arm have been reported [27, 28]. However, recent data show that adding ifosfamide and etoposide to high-dose methotrexate, cisplatin, and doxorubicin is associated with additional morbidity with no impact on survival outcome for the poor responders [29].

**Figure 2:**
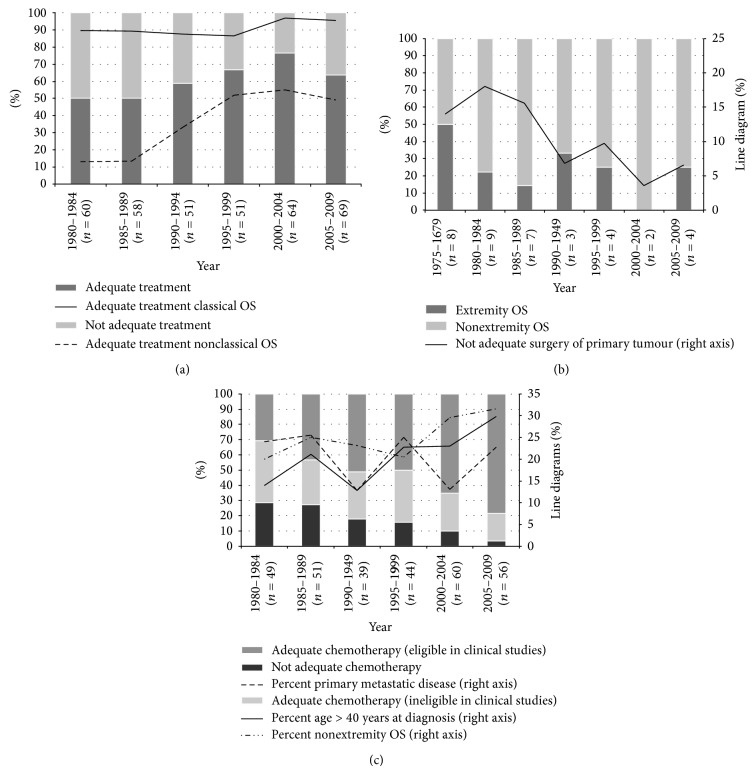
Time trends in distribution of osteosarcoma (OS) patients according to different subgroups: adequate versus not adequate primary treatment (both surgery and chemotherapy), including the percentage of adequate treatment among classical and nonclassical OS (a). The distribution of not adequate primary surgery among extremity versus nonextremity OS (b). Adequate versus not adequate chemotherapy. The percentage of patients >40 years of age at diagnosis, primary metastatic disease, and nonextremity tumour that received chemotherapy (c).

**Figure 3:**
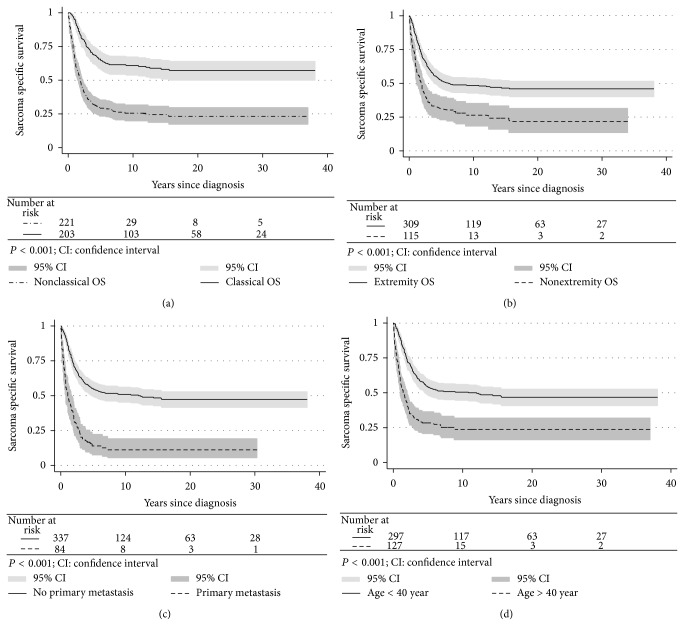
Sarcoma specific survival of classical versus nonclassical osteosarcoma (OS) patients (a). Extremity versus nonextremity OS (b). Patients with and without metastasis at time of diagnosis (c). Patients above and below 40 years of age at time of diagnosis (d).

**Figure 4:**
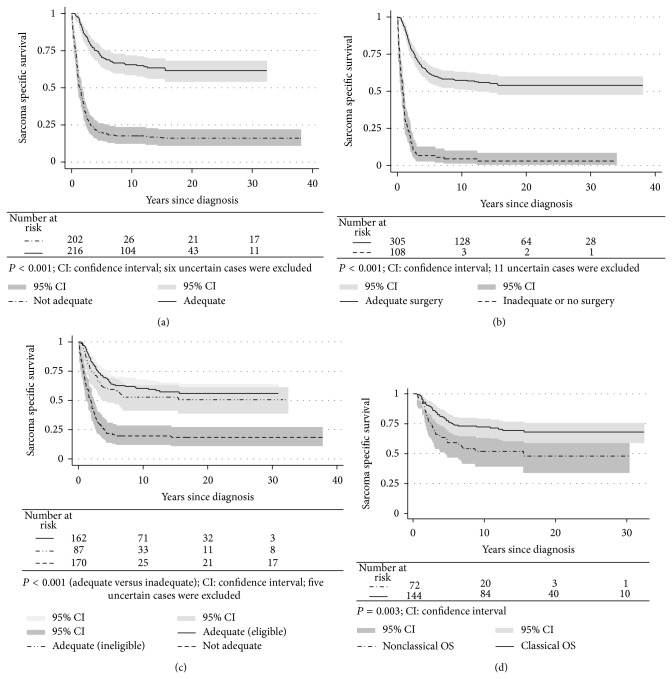
Sarcoma specific survival for osteosarcoma (OS) patients dependent on adequate primary treatment (both surgery and chemotherapy) (a); adequate surgery (b); adequate chemotherapy (c); adequate primary treatment among classical versus nonclassical OS (d).

*Adequate primary treatment* was defined as having received both adequate chemotherapy and adequate surgery. All treatment had to be given before metastatic disease or local relapse was verified among patients without primary metastatic disease. The below mentioned treatment variables were assessed both in relation to all patients that actually received chemotherapy (pre- and/or postoperative) and/or surgery and for all patients in the cohort regardless of given treatment or not (Tables 3–5 and Figures 2–4). We have not chosen to include radiotherapy in our definition of adequate primary treatment, since OS is known to be relatively resistant to such therapy [30]. However, there still may be a place for radiotherapy as local treatment of unresectable OS, following marginal or intralesional surgery, or as palliation of metastases [30–32].

*Adequate chemotherapy* was defined as receiving at least six courses of chemotherapy containing a minimum of two of the following drugs: high-dose methotrexate (at least 8 g/m^2^), doxorubicin, cisplatin, or ifosfamide in line with a previous definition by Sæter and Bruland [33]. These four drugs are most commonly used worldwide, even though there is still no international consensus on their optimal combination [9, 34]. We did not consider the CAMOS regimen [12] as adequate chemotherapy in this study, as compared to the current standard for active chemotherapy. We have not included histological response analysis in this study (see Section 4).

*Adequate surgery* implied surgical removal of primary tumour with wide or marginal margins [35], as defined by the surgeon and pathologist, and was always attempted. In this analysis we dichotomised between amputation (last surgery performed) and limb-sparing surgery, including 13 cases of rotationplasty. Patients with metastatic disease at time of diagnosis were in need for a complete surgical remission for both primary tumour and metastases, mostly to the lungs, in order to be classified as having received adequate surgery. However, in three cases we accepted just adequate surgery towards primary tumour when preoperative chemotherapy had “melted away” visible lung metastases present at time of diagnosis and with no thoracotomy performed.

### 2.4. Statistical Analyses

All prognostic variables were initially investigated by descriptive statistics. Relative risk (RR) and chi square analyses were applied. Survival analyses using Kaplan-Meier estimates [36], log rank tests [37], and Cox regression [38] were used to analyse overall survival, sarcoma specific survival (SSS), and event free survival (EFS). To identify the interactions between different prognostic factors, both univariate and multivariate Cox regression were applied. Overall survival was calculated from date of diagnosis [10] until death from any cause, while sarcoma specific death or treatment-related death was the endpoint of SSS. Patients with synchronous or metachronous skeletal osteosarcomas [39] were not censored in these analyses. EFS was calculated from date of diagnosis until the date of first metastasis, local recurrence, sarcoma specific death, or treatment-related death whichever occurred first. Patients with primary metastatic OS were not included in the analyses regarding EFS.

The endpoint for all survivors in this study was set to July 2013 using updated registries [10], to prevent bias due to nonidentical followup of patients with few or frequent appointments. The mean and median followup time for survivors were 18 and 17 years, respectively.

Statistically significant prognostic variables in univariate analysis were included into multivariate backward Cox-regression analyses. However, the three main subgroups of NCOS (Figure 1) were also included in the last step of the latter Cox model, independent of *P* values, to show their main estimated values and related confidence intervals. The Cox proportion hazard assumption was evaluated using Kaplan-Meier plots. The potential effect of missing values was evaluated using multiple MCMC imputation [40]. The statistical analyses were conducted using SPSS version 22 (SPSS Inc., Chicago, IL) and Stata version 13.1 (Stata corporation, College Station, TX).

### 2.5. Ethical Approval

The Regional Ethical Committee was informed, although the study did not require a formal ethical approval since the data registration was in line with the legitimate mandate of the NCR [10].

## 3. Results

### 3.1. Patient Characteristics

As shown in Table 1, 221 patients were NCOS (52%) and 203 were COS (48%). The main characteristics of these patients are also reported in Table 1, with a further description of the NCOS in Figure 1 and Table 2. The three overlapping ellipses in Figure 1 comprise age above 40 years at time of diagnosis, primary metastatic disease, or nonextremity localized primary tumour, each divided into four parts, with their associated numbers of patients. This is in order to illustrate the overlapping parts between the three subgroups of NCOS, for example, 127 patients above 40 years of age at time of diagnosis (45 + 14 + 17 + 51). However, only 45 of these patients had extremity localized primary tumour with no detectable metastasis at primary diagnoses. Similarly, 17 patients presented with all three elements of NCOS.

The male to female ratio in the classical group was 1.9 compared to 1.0 among the NCOS. The latter had about the same gender balance in its three groups. We observed a significantly higher rate of axial OS among patients above 40 years (Table 2), as previously reported [9, 10, 17]. As presented in Table 2, we neither identified any significant differences in the percentage of patients with primary metastatic disease dependent on OS in axial versus appendicular skeleton (RR = 1.0) nor due to age (RR = 1.3). Primary metastatic disease was most commonly in the lungs only (approximately 80%), otherwise developed in bone and lungs (six cases), bone only (ten cases), and one case with a soft tissue metastasis.

As demonstrated in Table 2, there were no significant differences in tumour size between COS and the total cohort of NCOS patients (RR = 1.1) in contrast to time to diagnosis (RR = 1.5). Patients with OS in the weight-bearing extremity skeleton had five times increased risk of a pathologic fracture compared to patients with an axial tumour (RR = 0.2). Also elderly patients had increased risk for a pathologic fracture (RR = 1.7). Patients with pathological fractures had slightly larger tumours (median 11 cm, range 21 cm) compared to all patients (median 10 cm, range 32 cm). The four cases of pathological fractures in the axial skeleton (Table 2) were all due to radiation-induced OS.

NCOS patients (all subgroups) had increased ALP levels compared to COS, while no corresponding significant differences were found regarding LDH, except for patients with primary metastatic disease (RR = 1.7).

### 3.2. Treatment-Overall Results

Table 3 outlines the extent of treatment administered to patients in the different groups of OS. 216 patients received adequate primary treatment from 1975 to 2009, and twice as many COS received adequate treatment compared to NCOS (Figure 4(d)), fully displayed in Table 6.

*COS*. Adequate surgery was performed in all except four cases (Table 3). Two patients had intralesional margin after surgery of humerus OS: one amputation and one limb sparing surgery, respectively. The latter patient received postoperative radiotherapy, 50 Gy. A third patient was wrongly classified as low grade OS, received no chemotherapy, and operated with a Kotz-prosthesis of femur, but with intralesional margin. The primary histology was later revised to high-grade OS when she was diagnosed with a local relapse and subsequently lung metastases. The fourth patient died of toxicity due to neoadjuvant chemotherapy before surgery was performed. Among the 56 COS patients not receiving adequate chemotherapy (28%), 39 were treated according to the previously mentioned CAMOS combination. From 1980 and onwards 91% of all COS received adequate treatment.

*NCOS*. In this group, only 33% (72 patients) received adequate treatment for the whole time period (Table 3) and 37% from 1980 to 2009. As expected, the reason for inadequacy was dominated by incomplete or absence of surgery in patients with primary metastatic disease or axial tumours, by inadequate chemotherapy in elderly patients, and by inadequacy of both modalities in patients with several factors combined (Table 3).

### 3.3. Treatment-Time Trends

*Adequate Primary Treatment*. The percentage of adequate treatment increased from 50% during the 1980s to 64% since 2005 (Figure 2(a)). We could also document an improved treatment during the 1990s for the NCOS group, with just over 50% adequate treatment since 1995, in contrast to slightly above 95% among COS patients since the millennium.

*Adequate Surgery*. For surgically treated patients we observed a reduction in the frequency of inadequate surgery of primary tumour from about 18% to approximately 5% since the millennium (Figure 2(b)). 27% of the inadequately treated primary tumours (10 patients) were related to a tumour in the appendicular skeleton. Four of these 10 patients had primary metastatic disease and five were above 40 years of age at time of diagnosis. Correspondingly, 27 patients with inadequate surgery had their primary tumour in the axial skeleton.

*Surgical Procedures*. Extremity OS patients were amputated during the 1970s (55 patients) with only one exception. We confirmed the significant increase in limb salvage procedures since the 1980s [5, 41–43], although amputation remains a valid procedure in selected cases of OS [44]. The percentage of amputation decreased from 89% during 1980–84 to 18% since the millennium. Since 1980, 97% of all surgical procedures for primary extremity tumours revealed adequate margins, with no significant differences between amputation and other surgical procedures (*P* = 0.235). Six cases with uncertain margins were excluded from this analysis. However, 69% of all patients in the amputated group had adequate combined treatment compared to 87% in the other group during 1980–2009 (*P* = 0.001). This difference was due to fewer patients with adequate chemotherapy in the first mentioned group. Hence, there was a significant difference in SSS between these two groups since 1980 (RR = 1.7, 95% CI 1.1–2.4, *P* = 0.011). No corresponding conclusion regarding adequately treated patients during 1980–2009 could be drawn, although RR was estimated to 1.5 (95% CI 0.9–2.5, *P* = 0.106).

*Adequate Chemotherapy*. We observed an increase in the percentage of OS patients with adequate chemotherapy from 1980–2009 (Figure 2(c)). Correspondingly, the percentage of patients given adequate chemotherapy and enrolled in prospective clinical studies increased from the 1980s and onwards. 119 patients (83%) in the COS group with adequate chemotherapy were included in the consecutive trials. 43 patients in the NCOS group (41%) were also included in clinical studies, that is, 15 in Euroboss 1 [25], 13 in EURAMOS 1 [25], 13 in ISG/SSG II [23], one in SSG VIII [21], and one in SSG II [20], respectively. The latter two cases were later excluded from these studies due to verified lung metastases just weeks after time of diagnosis. 42% of the 83 patients that received adequate chemotherapy and included in clinical trials since the millennium were NCOS.

Figure 2(c) also illustrates the improved time trends of chemotherapy to elderly patients and those with primary tumour in the axial skeleton from 1980 to 2009. No corresponding trend was seen among patients with primary metastatic disease (Figure 2(c)).

### 3.4. Metastatic Relapse or Local Recurrence during Followup

Among patients without primary metastatic disease, 42% of the nonextremity OS (39 patients) and 50% with extremity OS (124 patients) developed metastases during followup (*P* = 0.317). Approximately 90% developed lung metastases. Among patients with axial OS and no primary metastatic disease, 32% (29 patients) experienced local relapse during followup compared to 4% (9 patients) among extremity OS (*P* < 0.001). The median time to first metastatic event or local recurrence were 1.3 years (range 11.5 years) and 1.1 years (range 10.3 years) from diagnosis, respectively.

45% of the patients with local recurrence (17 patients) were never diagnosed with metastasis during followup and 16 were related to relapses in the axial skeleton (11 cases in the skull or jaw, four in costa, and one in the clavicle). 12 of these patients died due to their local relapse and without metastasis.

### 3.5. Survival Analyses

The survival rates in elderly people, patients with axial OS, or metastatic disease at time of diagnosis (Figures 3(b)–3(d)) were inferior to COS (Figure 3(a)). 10-year SSS for the latter group was 61% in contrast to 26% for the NCOS. As expected, patients who received adequate treatment had significantly better survival than inadequately treated patients (Figures 4(a)–4(c)). We also found a significant difference in survival among adequately treated COS versus NCOS patients (Figure 4(d)).

Patients enrolled in prospective studies (170 patients) had significantly better SSS than the 254 cases not formally included (RR = 0.4, 95% CI 0.3–0.6, *P* < 0.001). However, among the group that received both adequate surgery and chemotherapy (216 patients) we could not demonstrate any significant difference in SSS between the 148 patients formally included into clinical trials and the corresponding 68 patients treated according to the different protocols (RR = 1.1, 95% CI 0.7–1.8, *P* = 0.618).

### 3.6. Prognostic Factors

Table 4 presents the results of univariate analysis as ten-year overall survival and EFS according to different characteristics of OS including time periods and the main treatments variables. Histological subgroups (*P* = 0.167/0.435, log rank) and amputation versus other surgeries (*P* = 0.113/0.067, log rank) were not included in Table 4. We observed essentially the same positive prognostic factors influencing survival as shown in Table 2. All treatment variables presented had a positive and significant impact on outcome (Table 4).

Multivariate analyses of the prognostic factors significant by univariate analysis in Table 4 are presented in Table 5. We have not taken into account multiple imputation of missing values in this report, since their effects were considered as modest. Adequate primary treatment, elevated LDH, and decade of diagnosis were all significant factors for overall survival and EFS. Primary metastatic disease, age >40 years, and elevated tumour size were also adverse factors for overall survival, whereas axial tumour localisation did not reach significance (Table 5). Further, NCOS was an adverse factor for overall survival (RR = 2.0, 95% CI 1.4–2.9, *P* < 0.001).

Some main characteristics of the 216 patients receiving adequate primary treatment are displayed in Table 6. Female gender had a significantly improved overall survival among all adequately treated patients (RR = 1.9). We observed also just minor differences in RR between all patients with primary metastatic disease (RR = 3.1, Table 4) and those in this group that received adequate treatment (RR = 2.9, Table 6). Patients with either axial OS or age >40 years had a larger difference in RR, but the observed difference was not statistically significant with uncertainty due to the limited number of observations (data not shown).

## 4. Discussion

This population-based study provides reliable evidence that the overall outcome of an unselected cohort of patients with high-grade OS is less impressive than widely assumed (Figure 3 and Table 4). To our knowledge, no previous nationwide studies have in detail analysed the NCOS group, representing just over half of the patients in our cohort. We have by purpose not reported too detailed information regarding the clinical variables, in order to reduce the number of missing variables. Still, we believe that our key variables are valid to expand our knowledge regarding especially the NCOS population.

About 30% of our patients were above 40 years of age at time of diagnosis in line with previous nationwide studies [14, 16], although somewhat lower than reported by Whelan et al. [17]. As previously reported [10], just over one-fourth of all patients had an axial OS and one-fifth primary metastatic disease. For the latter group we found no differences among patients with primary tumour in the axial versus appendicular skeleton, in contrast to a previous report [45]. However, only 5% in the study of Bielack et al. had tumour located in the axial skeleton. This makes direct comparison difficult.

We confirmed a higher male to female ratio in extremity compared to nonextremity tumour [46]. Although gender had a significant effect on survival for adequately treated patients in the current study (Table 6), we identified no corresponding effect for all patients (Table 4). Nevertheless, there seems to be no unanimous answer regarding the relationship of sex on survival in the available literature [15, 16, 42, 47–50].

We found larger primary lesions among patients with primary metastatic disease (Table 2) in line with a previous study [51]. Patients with axial primary tumours had on average smaller lesions than patients with extremity localised tumours. This seems reasonable due to less “space” for tumour growth, for example, spine or head and neck region. Elevated tumour size was an independent prognostic factor for outcome (Table 5).

The presence of a pathologic fracture, typically in the weight-bearing extremity skeleton, was significantly higher among elderly patients (Table 2), despite a lower percentage of extremity OS among the elderly; this is in line with the literature [9, 10, 17]. Osteoporosis may be one contributing factor in this regard. Pathologic fracture has been considered as a poor prognostic factor [52] but did not reach significance in our analysis for the whole cohort (Table 4).

We found a dismal SSS among NCOS versus COS patients (Figure 3(a)). However, “less adequate” treatment could not explain the entire discrepancy in SSS between these two groups (Figure 4(d)). One important reason for the latter observation could be the poor prognosis for patients with primary metastatic disease, independent of given treatment (see below). In addition, nonextremity tumours have been associated with a higher rate of local recurrence than extremity OS, while no corresponding differences regarding first metastatic event were documented [46]. We confirmed these results in the present study. Both local recurrence and metastatic relapse are associated with rather poor prognosis [53–57], even though postrelapse survival seems to be higher among patients with late relapse [58].

Adequate primary treatment, regarding both surgery and chemotherapy, had a positive prognostic impact on survival in this study (Table 5). As previously reported [9, 10, 16], we also confirmed the improvement in overall survival since the late 1970s mainly due to the use of multiagent chemotherapy [9, 59].

We could not verify any stage migration over time due to better imaging [60] since we observed a fluctuation among patients with primary metastatic disease between 1975 and 2009. However, the “true” incidence could be confounded by low numbers of patients, approximately 2-3 cases annually within this time frame.

LDH was also an independent adverse prognostic factor in our study, in line with the literature [47, 61, 62]. However, we found no significant impact of ALP on outcome in the Cox analysis, in contrast to previous reports [63, 64].

Primary metastatic disease was a strong negative prognostic factor in our study (Tables 4–6), in line with the literature [14, 42, 51, 65–68]. The poor results may relate to primary chemotherapy resistance [8] partly due to different biologic characteristics of these tumours [51] and the numbers of metastases at diagnosis [69] besides the absence of complete surgical remission [69]. Hence, new therapeutic approaches at initial presentation are warranted for the primary metastatic OS patients [8, 65, 66, 70].

We observed just a minor adverse impact on survival of age >40 years among adequately treated patients (RR = 1.3) in contrast to all elderly patients in the cohort (RR = 2.7). It has earlier been advocated that patients above 40 years should, whenever possible, be treated similarly to those in younger age group [71]. Still, more than 77% of all elderly patients in this study received inadequate primary treatment from 1975–09, mainly due to inadequate chemotherapy (Table 3). Age >40 years at diagnosis was also a significant adverse factor in the multivariate analyses for overall survival but not for EFS (Table 5). Hence, more information regarding this group of patients, including their tolerance to chemotherapy, is warranted, for example, the results from the ongoing Euroboss 1 protocol [25].

It is well established that axial localisation results in worse outcome than primary disease arising in the appendicular skeleton [33, 46, 48, 72]. This is partly explained by a higher rate of axial tumours in the elderly patients. We have far too many cases with inadequate surgery among our patients with axial OS (Table 3 and Figure 2(b)), with local relapse as a frequent consequence.

Overall, we confirm primary metastatic disease, elevated tumour size, elevated serum LD, and old age as adverse prognostic factors in line with the literature [6, 7]. Neither elevated serum ALP nor axial primary tumour site were significantly adverse factors in the present study in contrast to previous findings [6, 7]. Nevertheless, differences in risk assessment might occur due to inequalities in, for example, methods adopted to identify and to analyse the various prognostic factors in different studies. The results may also depend on the clinical and demographic characteristics of the OS population analysed.

We cannot rule out that not including histologic response to preoperative chemotherapy may have affected the results in Table 5. Our decision was based on the different grading systems used from 1975 to 2009 [8], and the preoperative multidrug combinations have also changed considerably since the introduction by Rosen et al. [73]. These variables have already been fully published in the publications from the consecutive trials of the SSG (II, VIII, XIV) [20, 21, 24] and the ISG/SSGI [22]. More active drugs have also been added to the preoperative schedules and the time-line has been extended [8].

In addition, we could not evaluate a histological response to chemotherapy without a complete and uniform histological reexamination of all cases in the cohort. As of today only about 20% of all cases were formally reexamined histologically as part of this project [10]. However, a consequential and significant disadvantage of such an approach is the lack of available histological specimens for reexamination. For example, a previous Finnish study experienced a drop-out rate of 34% due to missing original specimens in such a setting [15]. Hence, we have chosen not to include histological response to chemotherapy in this paper since we believe the potential disadvantage will exceed the potential gain of such an approach.

Further improving cure rates for osteosarcoma continue to be challenging [1, 9, 74, 75]. To our knowledge, this is the first study addressing scopes beyond the classical OS patients based on a nationwide, population-based study. Differences in treatment could only partly explain the dismal outcome of NCOS patients as they present other tumour biological characteristics and clinical challenges compared to the COS.

## Figures and Tables

**Table 1:** Characteristics of nonclassical and classical osteosarcoma (OS) patients, 1975–2009.

	Nonclassical OS patients (%)	Classical OS patients (%)	All patients^a^ (%)
All patients	221 (52)	203 (48)	424 (100)

Gender			
Female	108 (49)	70 (34)	178 (42)
Male	113 (51)	133 (66)	246 (58)
Axial versus extremity			
Extremity	106 (48)	203 (100)	309 (73)
Axial	115 (52)	0	115 (27)
Primary metastatic disease			
No	134 (61)	203 (100)	337 (80)
Yes	84 (39)	0	84 (20)
Age			
≤40 years	94 (43)	203 (100)	297 (70)
>40 years	127 (57)	0	127 (30)
Tumour size			
≤10 cm	84 (52)	102 (56)	186 (54)
>10 cm	78 (48)	80 (44)	158 (46)
Duration of symptoms			
≤3 months	86 (45)	120 (62)	206 (53)
>3 months	107 (55)	73 (38)	180 (47)
Pathologic fracture			
No	187 (85)	178 (88)	365 (86)
Yes	34 (15)	25 (12)	59 (14)
ALP			
Normal	62 (42)	104 (68)	166 (55)
Elevated	87 (58)	48 (32)	135 (45)
LDH			
Normal	73 (51)	87 (61)	160 (56)
Elevated	70 (49)	56 (39)	126 (44)
Histology			
Osteoblastic	97 (44)	94 (47)	191 (46)
Chondroblastic	30 (14)	25 (13)	55 (13)
Fibroblastic	20 (9)	21 (11)	41 (10)
Other	73 (33)	59 (30)	132 (32)

^a^Missing values equal the difference between the summarized number from each subgroup in the fourth column and the total patients in the study.

**Table 2:** Relative risk analysis among nonclassical osteosarcoma (OS) patients; primary metastatic disease, nonextremity localisation, and age >40 years.

	Nonclassical OS		Primary metastatic disease			Nonextremity localisation			Age >40 years		
	RR^a^ (95% CI^b^)	*P* ^c^	*N* ^d^	RR^a^ (95% CI^b^)	*P* ^c^	*N* ^d^	RR^a^ (95% CI^b^)	*P* ^c^	*N* ^d^	RR^a^ (95% CI^b^)	*P* ^c^
Axial versus extremity											
Extremity			61	1		0			59	1	
Axial			23	1.0 (0.7–1.5)	0.953	115			68	3.4 (2.5–4.6)	<0.001
Primary metastatic disease											
No			0			89	1		94	1	
Yes			84			23	1.0 (0.7–1.5)	0.857	31	1.4 (0.9–2.1)	0.102
Age											
≤40 years			53	1		47	1		0		
>40 years			31	1.3 (0.9–1.8)	0.109	68	3.1 (2.4–4.1)	<0.001	127		
Tumour size											
≤10 cm	1		18	1		51	1		48	1	
>10 cm	1.1 (0.9–1.4)	0.436	38	1.6 (1.3–2.0)	<0.001	26	0.7 (0.5–1.0)	0.026	42	1.0 (0.8–1.3)	0.870
Duration of symptoms											
≤3 months	1		43	1		35	1		41	1	
>3 months	1.5 (1.2–1.8)	<0.001	34	0.9 (0.7–1.2)	0.632	61	1.6 (1.3–1.9)	<0.001	65	1.5 (1.2–1.8)	<0.001
Pathologic fracture											
No	1		68	1		111	1		102	1	
Yes	1.3 (0.8–2.0)	0.364	16	1.5 (0.9–2.4)	0.124	4	0.2 (0.1–0.5)	0.001	25	1.7 (1.1–2.8)	0.025
ALP											
Normal	1		18	1		34	1		34	1	
Elevated	1.9 (1.4–2.4)	<0.001	46	1.9 (1.5–2.4)	<0.001	43	1.4 (1.1–1.8)	0.017	47	1.5 (1.1–1.9)	0.003
LDH											
Normal	1		21	1		43	1		41	1	
Elevated	1.3 (1.0–1.6)	0.098	41	1.7 (1.4–2.2)	<0.001	31	0.9 (0.7–1.3)	0.667	37	1.1 (0.8–1.5)	0.473
Histology											
Osteoblastic	1		44	1		48	1		51	1	
Chondroblastic	1.0 (0.9–1.1)	0.622	8	1.1 (1.0–1.3)	0.124	20	0.9 (0.7–1.0)	0.135	18	0.9 (0.8–1.1)	0.404
Fibroblastic	1.0 (0.9–1.1)	0.816	8	1.0 (0.9–1.2)	0.610	9	1.0 (0.9–1.2)	0.656	13	1.0 (0.8–1.1)	0.530
Other	0.9 (0.8–1.1)	0.423	23	1.1 (0.9–1.4)	0.270	38	0.9 (0.8–1.2)	0.476	44	0.9 (0.7–1.1)	0.220

^a^Relative risk, ^b^confidence interval, ^c^ *P* value, and ^d^number of patients.

**Table 3:** Patients who received inadequate treatment dependent of subgroup of high-grade osteosarcoma (OS).

	Patients^a^	Not adequate treatment (%^b^)	Reason for inadequacy		
			Surgery (%^c^)	Chemotherapy (%^c^)	Both (%^c^)
Classical OS	200	56 (28)	0 (0)	52 (93)	4 (7)
Nonclassical OS	218	146 (67)	32 (22)	45 (31)	69 (47)
Nonextremity only	39	20 (51)	9 (45)	6 (30)	5 (25)
Primary metastasis only	46	23 (50)	15 (65)	2 (9)	6 (26)
Age >40 only	45	32 (71)	0 (0)	24 (75)	8 (25)
Several factors combined	88	71 (81)	8 (11)	13 (18)	50 (71)

^a^Six uncertain cases were not included. ^b^In % of cases from second column. ^c^In % of all cases of inadequate treatment for each subgroup.

**Table 4:** Univariate Kaplan-Meier and Cox-regression analysis of ten-year overall survival and event-free survival according to different characteristics of all high-grade osteosarcoma.

	Overall survival				Event-free survival			
	Patients	10 years in %	RR^b^	*P* ^c^	Patients	10 years in %	RR^b^	*P* ^c^
	(%)	(95% CI^a^ in %)	(95% CI^a^)		(%)	(95% CI^a^ in %)	(95% CI^a^)	
Year of diagnosis				0.010				0.003
1975–1979	64 (15)	27 (17–38)	1.7 (1.2–2.4)		59 (17)	27 (16–39)	2.0 (1.3–3.0)	
1980–1989	121 (29)	36 (27–44)	1.6 (1.1–2.1)		91 (27)	33 (24–43)	1.7 (1.2–2.4)	
1990–1999	103 (24)	46 (36–55)	1.2 (0.8–1.6)		81 (24)	43 (32–54)	1.4 (0.9–2.0)	
2000–2009	136 (32)	47 (37–56)	1		109 (32)	49 (39–59)	1	
Gender				0.605				0.433
Female	178 (42)	43 (36–51)	1		134 (39)	45 (37–53)	1	
Male	246 (58)	39 (32–45)	1.1 (0.8–1.4)		206 (61)	36 (30–43)	1.1 (0.8–1.5)	
Axial versus extremity				<0.001				<0.001
Extremity	309 (73)	47 (41–53)	1		248 (73)	46 (39–52)	1	
Axial	115 (27)	23 (16–32)	2.1 (1.6–2.7)		92 (27)	25 (16–34)	2.1 (1.6–2.8)	
Primary metastasis				<0.001				
No	337 (80)	48 (43–54)	1					
Yes	84 (20)	11 (6–19)	3.1 (2.4–4.1)					
Age				<0.001				<0.001
≤40 years	297 (70)	50 (44–56)	1		244 (72)	47 (41–53)	1	
>40 years	127 (30)	18 (12–26)	2.7 (2.1–3.5)		96 (28)	21 (13–30)	2.5 (1.8–3.3)	
Tumour size				0.005				0.035
≤10 cm	186 (54)	50 (43–57)	1		168 (58)	47 (39–54)	1	
>10 cm	158 (46)	38 (30–45)	1.5 (1.1–2.0)		120 (42)	35 (26–45)	1.4 (1.0–1.9)	
Duration of symptoms				0.542				0.444
≤3 months	206 (53)	45 (38–52)	1		163 (53)	42 (34–49)	1	
>3 months	180 (47)	38 (31–45)	1.1 (0.8–1.4)		146 (47)	38 (30–46)	1.1 (0.8–1.5)	
Pathologic fracture				0.119				0.492
No	365 (86)	42 (37–47)	1		297 (87)	40 (35–46)	1	
Yes	59 (14)	34 (22–46)	1.3 (0.9–1.8)		43 (13)	36 (22–51)	1.2 (0.8–1.7)	
ALP				<0.001				0.001
Normal	166 (55)	51 (43–58)	1		148 (62)	49 (40–57)	1	
Elevated	135 (45)	26 (18–33)	2.0 (1.5–2.7)		89 (38)	29 (20–39)	1.8 (1.3–2.4)	
LDH				<0.001				0.020
Normal	160 (56)	48 (40–56)	1		85 (38)	47 (39–55)	1	
Elevated	126 (44)	28 (20–36)	1.7 (1.3–2.3)		139 (62)	30 (20–40)	1.5 (1.1–2.1)	
Adequate chemotherapy				<0.001				<0.001
Yes	249 (59)	57 (50–63)	1		198 (58)	56 (48–62)	1	
No	170 (40)	17 (12–23)	3.4 (2.7–4.4)		137 (40)	17 (11–24)	3.9 (3.0–5.2)	
Uncertain	5 (1)	40 (5–75)	1.2 (0.4–3.9)		5 (1)	40 (5–75)	2.1 (0.8–5.7)	
Adequate surgery				<0.001				<0.001
Yes	305 (72)	55 (49–60)	1		277 (81)	48 (41–53)	1	
No	108 (25)	4 (2–10)	7.0 (5.4–9.2)		53 (16)	6 (2–14)	4.9 (3.5–6.8)	
Uncertain	11 (3)	9 (1–33)	2.9 (1.5–5.6)		10 (3)	0	3.4 (1.8–6.6)	
Adequate primary treatment				<0.001				<0.001
Yes	216 (51)	65 (58–71)	1		188 (55)	58 (50–65)	1	
No	202 (47)	15 (10–21)	4.8 (3.7–6.2)		147 (43)	18 (12–24)	4.3 (3.2–5.7)	
Uncertain	6 (2)	29 (4–61)	2.0 (0.7–5.5)		5 (2)	17 (1–52)	2.8 (1.1–6.9)	
Formal inclusion in trial				<0.001				<0.001
Yes	170 (40)	59 (51–66)	1		144 (42)	55 (47–63)	1	
No	254 (60)	28 (23–34)	2.4 (1.9–3.2)		196 (58)	29 (22–35)	2.5 (1.8–3.3)	

^a^Confidence interval, ^b^relative risk, and ^c^log rank test.

**Table 5:** Multivariate Cox-regression analysis of prognostic factors and treatment-related variables for overall survival and event-free survival. All high-grade osteosarcomas.

Variables^a^	Overall survival		Event-free survival	
	RR^b^	*P* ^d^	RR^b^	*P* ^d^
	(95% CI^c^)		(95% CI^c^)	
Adequate primary treatment				
Yes	1		1	
No	3.6 (2.3–5.4)	<0.001	6.6 (3.9–11.1)	<0.001
Uncertain	5.0 (1.2–21.8)	0.031	5.5 (1.3–23.5)	0.022
Elevated LDH	1.5 (1.1–2.1)	0.015	1.7 (1.2–2.4)	0.002
Primary metastatic disease	2.3 (1.5–3.4)	<0.001		
Year of diagnosis				
1975–1979	1.5 (0.8–2.8)	0.195	0.7 (0.4–1.4)	0.315
1980–1989	2.1 (1.3–3.2)	0.002	2.0 (1.2–3.2)	0.005
1990–1999	1.7 (1.1–2.7)	0.023	1.7 (1.0–2.8)	0.048
2000–2009	1		1	
Age >40 years	1.5 (1.0–2.2)	0.041	1.0 (0.6–1.5)	0.930
Tumour size >10 cm	1.4 (1.0–2.0)	0.046		
Axial primary tumour	1.4 (0.9–2.2)	0.124	1.1 (0.7–1.8)	0.587

^a^Reference values in line with Table 4. ^b^Relative risk, ^c^confidence interval, and ^d^ *P* value.

**Table 6:** Univariate Cox-regression analysis of overall survival among all adequate treated high-grade osteosarcoma patients.

	Patients (%)	RR^a^ (95% CI^b^)	*P* ^c^

Gender			
Female	84 (39)	1	
Male	132 (61)	1.9 (1.1–3.0)	0.012
Axial versus extremity			
Extremity	183 (85)	1	
Axial	33 (15)	1.3 (0.7–2.2)	0.455
Primary metastatic disease			
No	188 (87)	1	
Yes	28 (13)	2.9 (1.7–4.8)	<0.001
Age			
≤40 years	187 (87)	1	
>40 years	29 (13)	1.3 (0.7–2.3)	0.402

^a^Relative risk, ^b^confidence interval, and ^c^ *P* value.
